# An assessment of silver copper sulfides for photovoltaic applications: theoretical and experimental insights†
†Electronic supplementary information (ESI) available. See DOI: 10.1039/c6ta03376h



**DOI:** 10.1039/c6ta03376h

**Published:** 2016-07-23

**Authors:** Christopher N. Savory, Alex M. Ganose, Will Travis, Ria S. Atri, Robert G. Palgrave, David O. Scanlon

**Affiliations:** a University College London , Kathleen Lonsdale Materials Chemistry , Department of Chemistry , 20 Gordon Street , London WC1H 0AJ , UK . Email: d.scanlon@ucl.ac.uk; b Diamond Light Source Ltd. , Diamond House , Harwell Science and Innovation Campus , Didcot , Oxfordshire OX11 0DE , UK; c University College London , Department of Chemistry , London WC1H 0AJ , UK

## Abstract

As the worldwide demand for energy increases, low-cost solar cells are being looked to as a solution for the future. To attain this, non-toxic earth-abundant materials are crucial, however cell efficiencies for current materials are limited in many cases. In this article, we examine the two silver copper sulfides AgCuS and Ag_3_CuS_2_ as possible solar absorbers using hybrid density functional theory, diffuse reflectance spectroscopy, XPS and Hall effect measurements. We show that both compounds demonstrate promising electronic structures and band gaps for high theoretical efficiency solar cells, based on Shockley–Queisser limits. Detailed analysis of their optical properties, however, indicates that only AgCuS should be of interest for PV applications, with a high theoretical efficiency. From this, we also calculate the band alignment of AgCuS against various buffer layers to aid in future device construction.

## Abstract 2

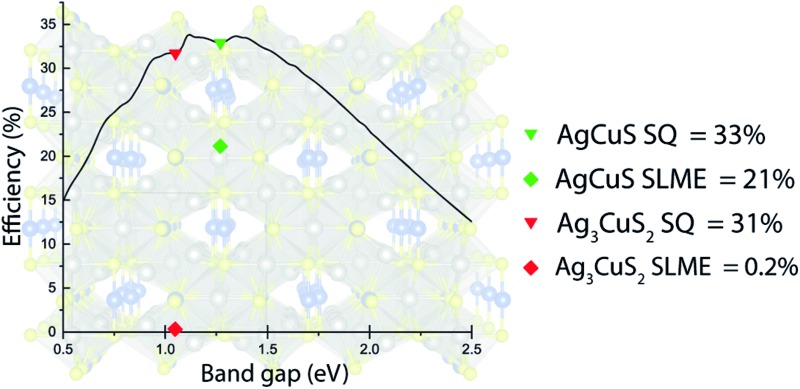
As the worldwide demand for energy increases, low-cost solar cells are being looked to as a solution for the future.

## 1 Introduction

The photovoltaic (PV) industry has grown rapidly in the past decade to meet an ever-rising demand for energy that avoids dependence on fossil fuel technology; the importance of such technology is demonstrated by the production of PV devices, which has increased by 40% each year from 2000 to 2012.^1^ To meet this demand it is crucial that the materials used in these devices are as efficient and cost effective as possible in order to ensure widespread availability. The current material of choice for photovoltaics is crystalline silicon, which benefits from being abundant and having been optimized over the course of half a century to high efficiencies;^2^ however, it also suffers from high energy consumption in the growth of silicon boules, causing a relatively long energetic payback time,^3^ its inherent inefficiency due to its indirect fundamental band gap, and silicon wafers requiring significant thickness (>100 μm) in order to absorb sufficient light, increasing material consumption.

Thin-film materials such as CdTe, GaAs and Cu(In,Ga)Se_2_ (CIGS) have seen an increase in market share within the last decade as cell efficiencies have increased with optimization: up to 21% for CdTe and CIGS, and 28.8% for GaAs.^4^ In all these cases, they possess direct band gaps, leading to higher absorption and allowing for much thinner layers of material for the same cell efficiency as Si. They also boast a much lower energetic cost than silicon,^3^ together with band gaps closer to the optimal theoretical limit for a single junction cell predicted by Shockley and Queisser^5^ – the highest power conversion efficiencies for such a cell are possible between 1.0 and 1.7 eV and a maximum around 1.3 eV. Thin film absorbers do have their own problems however, with CIGS restricted by the low relative abundances of indium and gallium, and cadmium and arsenic's toxicity presenting a significant barrier to worldwide application of such technologies.

As such, there has been a recent drive to develop earth-abundant, non-toxic alternative photovoltaic materials, such as the antimony and bismuth copper chalcogenides, Cu_3_BiS_3_, CuSbS_2_ and CuSbSe_2_,^6–9^ the zinc tin pnictides, ZnSnN_2_ and ZnSnP_2_,^10–12^ and the binary antimony chalcogenides, Sb_2_S_3_ and Sb_2_Se_3_;^13,14^ despite having been shown to have suitable band gaps within the optimal 1.0–1.7 eV range, experimental cell efficiencies for these materials remain low.^15–18^ The current leader in this field is Cu_2_ZnSn(S,Se)_4_ (CZTSSe) which has a record cell efficiency of 12.6%, a tunable band gap of 1.0–1.5 eV, and is solution processable,^19–21^ however, cell efficiencies have since plateaued with few major advances in efficiency since 2013 as CZTS cells have been limited by a large deficit in open-circuit voltage compared to the band gap.^22–24^ The kesterite system also has complex defect physics due to its quaternary nature, meaning close control of Cu and Zn proportions, as well as inclusion of Na, is often critical for suppressing non-radiative recombination and attaining high efficiency cells.^25–30^

As a result, we look towards the simpler ternary silver copper sulfides, AgCuS (stromeyerite) and Ag_3_CuS_2_ (jalpaite). The silver copper sulfides have been examined historically for their ionic conductivity at high temperatures, and a number of studies have examined their phase behaviour.^31,32^ The most recent structural studies on the room temperature phases of these two compounds have been performed by Baker *et al.* ^33,34^ and Trots *et al.* ^35,36^ using single crystal X-ray and neutron powder diffraction. Previous theoretical investigations of AgCuS have focused on its high temperature cubic phase^37^ and its behaviour under pressure;^38^ the most recent work, however, showed that it exhibits p-type conductivity with a significant thermopower (∼665 μV K^–1^) at room temperature.^39^ Ag_3_CuS_2_ has previously, in combination with Ag_2_S and Ag, been shown to exhibit photocatalytic behaviour,^40^ but it is of immediate interest due to the recent publication of two solar cells utilizing it as an absorber layer.^41,42^

**Fig. 1:**
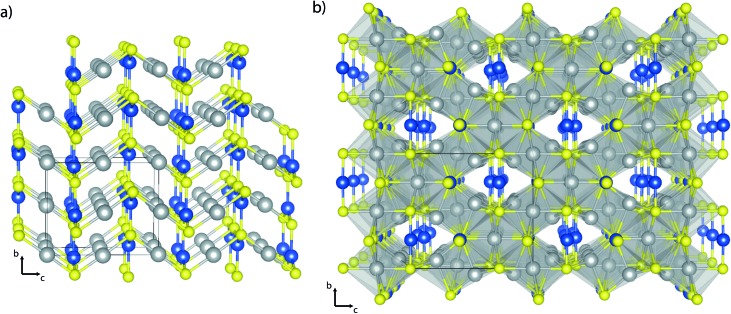
Crystal structures of (a) RT-AgCuS and (b) RT-Ag_3_CuS_2_. A single unit cell is marked in each, and the following atom labels are used: Ag atoms in grey, Cu in blue and S in yellow.

In this article, we examine the silver copper sulfides using hybrid density functional theory (DFT) with an aim to critically assess their suitability as photovoltaic absorber materials. Two different structures of the silver copper sulfides were investigated using DFT: the room temperature, or *β*, *Cmc*2_1_ phase of AgCuS,^33^ and the room temperature *I*4_1_/*amd* phase of Ag_3_CuS_2_.^36^ These structures are depicted in Fig. 1. Both of the structures studied are connected in all 3 dimensions: RT-AgCuS consists of zig–zag Ag–S chains, with the silver atoms linearly coordinated, which are bridged along a and b by 3-coordinate copper atoms. RT-Ag_3_CuS_2_ contains two different silver environments – octahedrally coordinated, and highly distorted tetrahedrally coordinated to sulfur; these environments are face-sharing and create the ‘X’ shaped structure seen in the {011} plane in Fig. 1b, while the copper atoms are linearly coordinated and bridge the channels in the structure. Different copper coordination environments have previously been observed to impact upon electronic properties in semiconductors, so the differences between these two structures are of interest.^43^ These structures were optimized computationally using a hybrid exchange–correlation functional, and the relative accuracy compared to experiment was assessed. From this, we present a thorough exploration of the optical and electronic properties of the systems of interest, with attention to how these may affect their photovoltaic behaviour.

## 2 Methods

### 2.1 Theoretical

Each structure in this report was optimized and electronic structure calculated using periodic DFT using the Vienna Ab Initio Simulation Package (VASP), which implements all the DFT and hybrid DFT functionals mentioned in this report.^44–47^ The primary functional used was the hybrid functional HSE06.^48^ This incorporates 25% Hartree Fock exchange in addition to 75% exchange from the Generalised Gradient Approximation (GGA) functional, PBE;^49^ additionally in HSE06, the HF exchange is screened using a parameter of *ω* = 0.11 bohr^–1^ such that it is only significant at short range. The projector-augmented wave method was used to account for valence and core electron interactions.^50^ A cutoff energy of 350 eV, a *k*-mesh spacing of 0.04 Å^–1^ along each reciprocal vector and a convergence criterion of 0.01 Å^–1^ on the forces per atom were used in all calculations. By utilising a hybrid functional, we hope to avoid the well-known problem with GGA-based DFT methods: that they can severely underestimate semiconductor band gaps.^51^ Also, we might expect that hybrid functionals, by including correct Hartree–Fock electron exchange, will also avoid some of the self-interaction error inherent in DFT calculations, which becomes particularly significant in systems which contain strongly correlated d electrons, like the silver copper sulfides. HSE06 was chosen as it has been shown to give accurate measurements of semiconductor band gaps in comparison to experiment in work on other semiconductor systems.^8,52–55^ To calculate the optical properties of the system, the method developed by Furthmüller *et al.* ^56^ was used to calculate the high-frequency dielectric function, from which the absorption coefficient can be derived. The valence band alignment of AgCuS was performed using the core-level alignment approach developed by Wei and Zunger.^57^ All crystal structures in this report were drawn in the VESTA visualisation program.^58^

### 2.2 Experimental section

#### Synthesis of AgCuS

AgCuS was synthesised using the hydrothermal method proposed by Tokuhara *et al.* ^59^ Non-stoichiometric quantities of Ag (1.4314 g, 0.0133 mol), Cu (1.0306 g, 0.0162 mol), and S (0.4727 g, 0.0147 mol), using a molar ratio of 0.9 : 1.1 : 1.0, were ground together in an agate pestle and mortar. Of this mixture 0.5 g was transferred to a Teflon-lined steel autoclave (45 mL) together with 15 mL distilled water. The reaction vessel was oven heated at 180 °C for 10 h, before being cooled slowly to room temperature. The AgCuS was isolated in quantitative yield *via* filtration; it was washed several times with distilled water and dried.

#### Synthesis of Ag_3_CuS_2_

Ag_3_CuS_2_ was synthesised from a non-stoichiometric molar ratio mixture of 3.05 : 1.00 : 2.00 of elemental Ag (1.6039 g, 0.0143 mol), Cu (0.3000 g, 0.0047 mol) and S (0.3027 g, 0.0094 mol). The reagents were ground together in an agate pestle and mortar and added to a 1 cm diameter quartz tube. The tube was evacuated and flame sealed before heating at 5 °C min^–1^ to 500 °C with a dwell time of 10 h followed by cooling slowly to room temperature. Ag_3_CuS_2_ was obtained in good yield without further washing or purification.

#### Characterisation

Powder X-ray diffraction (PXRD) data was collected on a Stoe StadiP diffractometer using Cu Kα1 (*λ* = 1.54056 Å) radiation. 0.5 mm capillaries were filled with powdered samples and data were collected over the 2*θ* range 5–60° in steps of 0.5° at 20 s per step. Optical diffuse-reflectance data was recorded between 300 and 2000 nm, with a data collection step of 1 nm, using a Lambda 950 spectrophotometer equipped with an integrating sphere at ambient temperature. AgCuS and the Ag_3_CuS_2_ were pressed into 13 mm diameter pellets at 5 bar with thicknesses of 1.04 mm and 1.24 mm for AgCuS and Ag_3_CuS_2_ respectively. The Hall coefficient, electrical resistivity, carrier concentration and carrier mobility of the AgCuS pellet were measured using van der Pauw geometry on a Ecopia Hall Measurement System (HMS-3000) at room temperature using four silver paint contacts. Four point probe measurements were performed, giving sheet resistance for Ag_3_CuS_2_, as resistance was too high for Hall effect measurement. X-ray photoemission spectroscopy (XPS) measurements were recorded using a Thermo Scientific Al-Kα.

## 3 Results and discussion

### 3.1 AgCuS

**Fig. 2:**
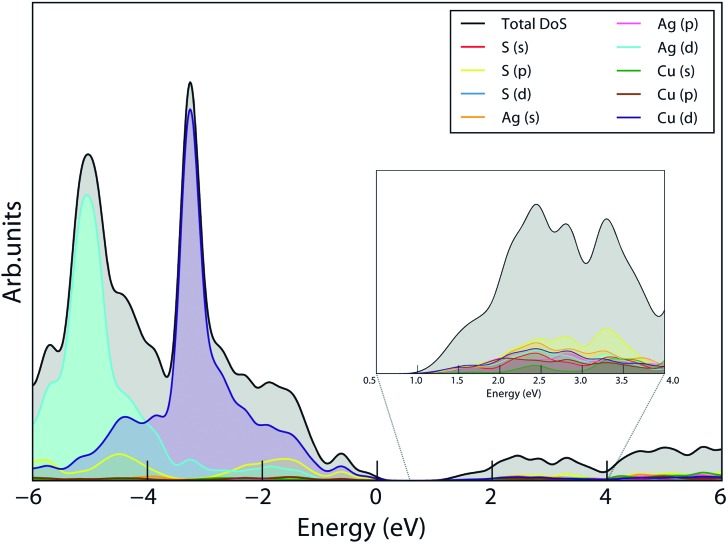
Total and partial density of states (DoS) of AgCuS, with HSE06; individual partial DoS are labelled in legend, valence band maximum (VBM) set to 0 eV.

Firstly, AgCuS was considered: calculated lattice parameters of *a* = 4.042 Å, *b* = 6.752 Å and *c* = 8.431 Å were obtained; a comparison of this geometry optimization with experiment is shown in the ESI (ST1†). The total and partial Density of States (DoS) diagram is shown in Fig. 2, demonstrating that the valence band is primarily composed of Ag d, Cu d and S p states, while Cu d and S p states also dominate the conduction band minimum, with some Ag s contribution. The additional localisation of the strongly correlated Cu d and Ag d states in HSE06 due to its partial correction of self-interaction error causes the bulk of these to be low in energy and leading to a large proportion of S p states at the valence band edge. A similar shift is seen in other Cu(i) and Ag systems.^60,61^

**Fig. 3:**
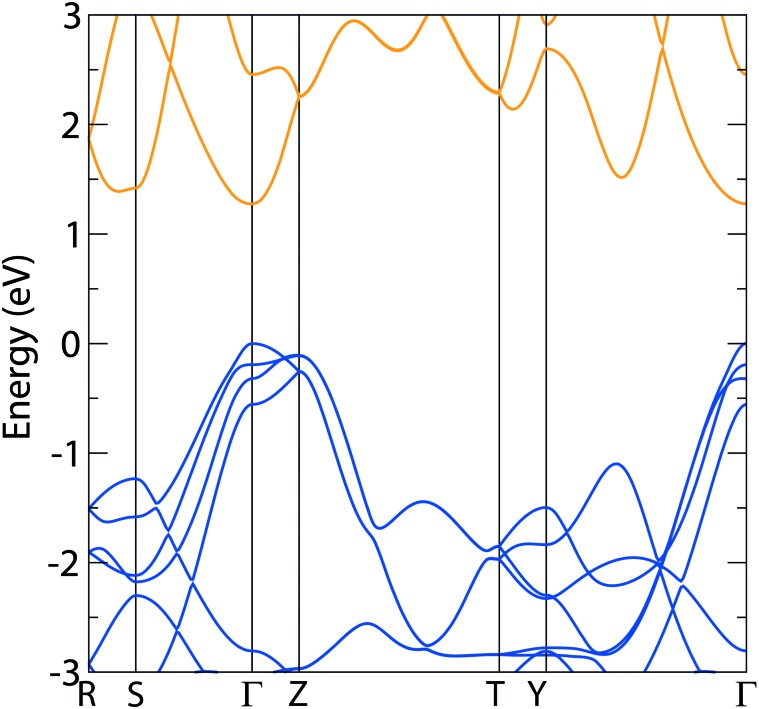
Band structure diagram of AgCuS using the HSE06 functional, showing a direct 1.27 eV band gap; valence band marked in blue, conduction band marked in orange, VBM set to 0 eV.

The HSE06 electronic band structure of AgCuS is shown in Fig. 3. The HSE06 band gap is direct, with a predicted value of 1.27 eV, well within the ideal range for photovoltaics, and corresponds to a maximum theoretical efficiency of around 33% under AM 1.5 illumination in the Shockley–Queisser (SQ) limit. Comparing to experimental work done by Guin *et al.*,^39^ which found an approximate band gap of 0.9 eV using optical diffuse reflectance measurements, this HSE06 result appears to be an overestimation. The band structure shows very significant dispersion in the valence band, and also a similar degree of dispersion in the conduction band. The resultant effective masses have been calculated from the HSE06 band structure and are listed in Table 1, showing that there is some anisotropy in the valence band effective masses, that they are particularly low (<0.4*m*_0_, indicating the possibility of high mobility^62^) along the copper–sulfur layers in the (001) plane, and they are close to those predicted in the kesterites, like CZTS.^63^ The electron effective mass is on average higher than that of the holes which, while unusual, has been seen in other photoabsorbers such as methylammonium lead iodide and BiSI.^54,64^ The magnitudes of *m*_e_ are also similar to those of other promising Cu-based photovoltaic absorbers.^6^

**Table 1:** Calculated effective masses of AgCuS and *I*4_1_/*amd* Ag_3_CuS_2_ from HSE06 band structures

	Valence band (*m*_0_)			Conduction band (*m*_0_)		
AgCuS	Γ → S	Γ → Y	Γ → Z	Γ → S	Γ → Y	Γ → Z
	0.311	0.242	1.795	1.362	0.736	0.493

	Valence band (*m*_0_)			Conduction band (*m*_0_)		
Ag_3_CuS_2_	Γ → N	Γ → X	Γ → Z	Γ → N	Γ → X	Γ → Z
	0.522	0.598	0.279	0.281	0.322	0.206

### 3.2 Ag_3_CuS_2_

The results from the structural optimizations of the *I*4_1_/*amd* phase of Ag_3_CuS_2_ are displayed in ESI Table 2,† with the calculated lattice parameters of *a* = 8.835 Å and *c* = 11.801 Å.

**Fig. 4:**
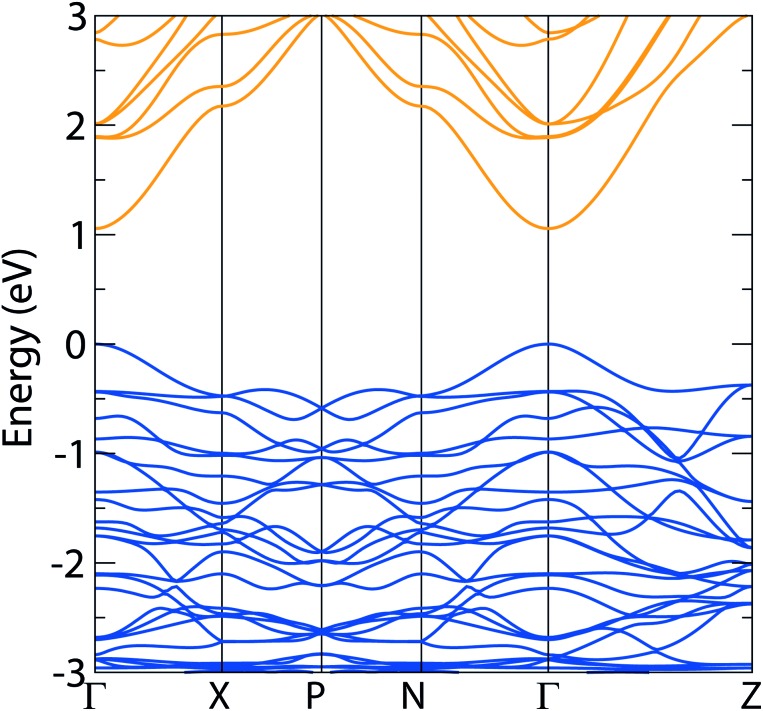
Band structure diagrams of *I*4_1_/*amd* Ag_3_CuS_2_ using HSE06, demonstrating a direct 1.05 eV band gap; valence band marked in blue, conduction band marked in orange, VBM set to 0 eV.

The density of states diagram for Ag_3_CuS_2_ shows similar trends to those seen in AgCuS: the same states make up the conduction and valence bands, albeit with a greater concentration of Ag d states due to the stoichiometry of the system, and localization of the Ag and Cu valence states within the valence band, resulting in a high DOS there – the density of state diagram for Ag_3_CuS_2_ is enclosed in the ESI (Fig. SF2†). At the valence band maximum, unlike AgCuS, the Ag d states dominate in proportion over the Cu d, although the bulk of the Ag d states remain lower in energy. The HSE06 band structure is displayed in Fig. 4. The most significant result from this is the direct fundamental band gap of 1.05 eV, which is encouraging, as it corresponds to a SQ limit of around 30%, well within the suitable range for PV, and is consistent with Ag_3_CuS_2_'s observed photoactivity.^41^ The band structure also demonstrates good dispersion in both the conduction and valence band, similar to that seen in AgCuS, indicating significant electron and hole mobility through the 3D structure. The effective masses for electrons and holes were calculated from the band structure, and are shown in Table 1; with average values of 0.270*m*_0_ and 0.466*m*_0_ respectively, these are comparable to other photovoltaic materials demonstrating high carrier mobilities.^65,66^

### 3.3 Synthesis and experimental analysis

**Fig. 5:**
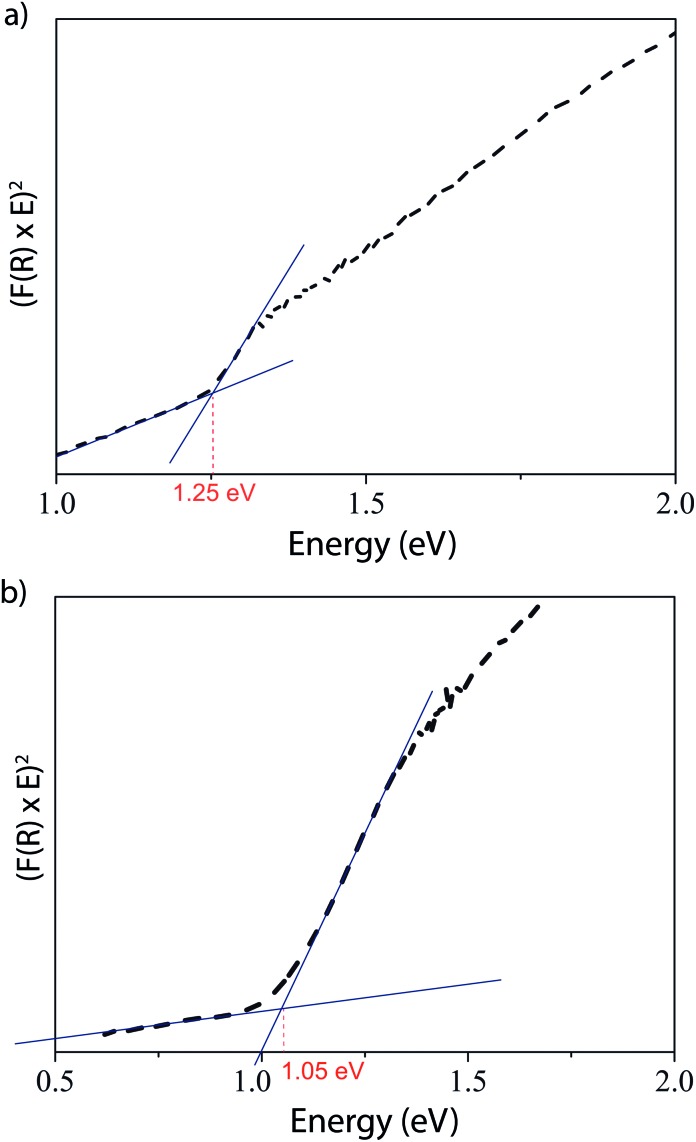
Kubelka–Munk plot from diffuse reflectance measurement of (a) AgCuS and (b) Ag_3_CuS_2_. Intersections of background and absorption marked, giving the optical band gaps, in red.

In addition to this theoretical work, experimental work was carried out to synthesise and characterise both compounds, and to verify some of the theoretical predictions above. Both compounds were synthesised from a mixture of their constituent elements: AgCuS was obtained using a hydrothermal method similar to that used by Tokuhara *et al.*,^59^ while Ag_3_CuS_2_ used a typical high temperature synthesis from the elements. In both cases, the products were obtained as black powders, and identified using powder X-ray diffraction; the resulting patterns were indexed in previously obtained space groups of *Cmc*2_1_ for AgCuS and *I*4_1_/*amd* for Ag_3_CuS_2_,^33,34^ and are shown in comparison to patterns simulated from those previous structures in ESI Fig. SF5.† Lattice parameters obtained by least squares refinement of the powder XRD peak positions were *a* = 4.0623(1) Å, *b* = 6.6254(2) Å, *c* = 7.9692(2) Å for AgCuS, and *a* = 8.6370(2) Å, *c* = 11.7688(5) Å for Ag_3_CuS_2_. A good match between the simulated and experimental patterns (no more than 0.2% difference in any lattice parameter), with a lack of impurity peaks, indicates that the powders obtained were single-phase and sufficiently pure for further analysis. Williamson Hall plots were used to estimate the volume weighted mean crystallite size. For AgCuS, the mean size was 50 nm, while for Ag_3_CuS_2_ the mean size was 70 nm; the larger size is consistent with the high temperature synthesis route used. Optical reflectance measurements were then performed on the samples to assess the experimental band gap; the resultant Kubelka–Munk plots are shown in Fig. 5. The experimental optical band gaps observed, 1.25 and 1.05 eV for AgCuS and Ag_3_CuS_2_ respectively, correlate very well with our predicted direct fundamental band gaps of 1.27 and 1.05 eV from the HSE06 calculations. A range of optical band gaps have been reported for AgCuS from 0.9–1.2 eV (ref. 39 and 67) with larger band gaps associated with small particle sizes. Our band gap value is at the larger end of this range, which is more in-keeping with our calculated value.

X-ray photoelectron spectroscopy (XPS) was also carried out on both materials. Core-level XPS, which demonstrates that Ag, Cu and S are present in both samples with no impurity states, is included in ESI Fig. SF6 and SF7.† As presented, both AgCuS and Ag_3_CuS_2_ samples gave XPS survey spectra indicating the presence of Cu, Ag, S and O, as well as adventitious carbon on the surface. In both cases etching with 2 keV Ar^+^ ions for 100 s was sufficient to totally remove the oxygen to below the detection limit of the instrument (*ca.* 0.5 atomic%). After etching the measured surface composition of the AgCuS sample was Ag_0.93_CuS_0.96_ and that of the Ag_3_CuS_2_ sample was Ag_3.05_Cu_1.03_S_2.01_. Given the usually quoted XPS composition error of up to 10%, these compositions are consistent with the expected formulae. The high resolution spectra discussed below all refer to the etched samples.

For both AgCuS and Ag_3_CuS_2_, the Ag 3d high-resolution XPS scans in (a) of both Fig. S6 and S7† show the expected spin orbit doublet with symmetrical peaks, the Ag 3d_5/2_ appearing at 368.2 eV in both compounds. These values are consistent with data reported by Chowdari *et al.* for Ag_2_S,^68^ and together with the absence of loss features which would be observed at the high binding energy side of the core line peaks if Ag metal were present indicate that Ag^+^ is the only detectable Ag species by XPS. Fig. S6 and S7† (b) show the Cu 2p high resolution scans for AgCuS and Ag_3_CuS_2_, with Cu 2p_3/2_ peaks at 932.5 eV (AgCuS) and 932.7 eV (Ag_3_CuS_2_) corresponding to known values for Cu_2_S.^69,70^ The Cu 2p peak shape is highly sensitive to Cu oxidation state, with Cu_2_^+^ states displaying strong satellite features, and Cu metal showing asymmetry due to plasmon energy loss processes. Therefore the symmetric Cu 2p peaks observed here, and the absence of any satellite peaks, indicates that Cu^+^ is the only copper oxidation state detected. The spin orbit components of the S 2p doublet are resolved for both compounds ((c) in Fig. S6 and S7†), with the S 2p_3/2_ peak measured at a binding energy of 161.5 eV in each case, consistent with expectations for S^2–^ ions.^69^ After etching, no higher binding energy S 2p peaks, corresponding to sulfate species, were observed, indicating that any oxidation is limited to the surface.

**Fig. 6:**
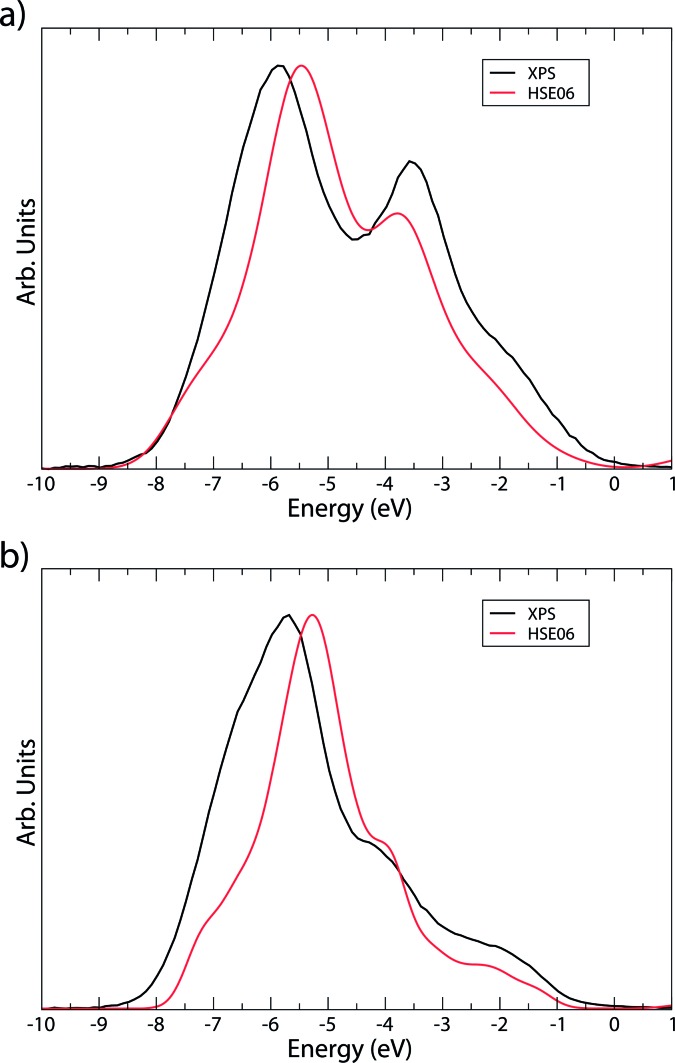
Comparison of valence band XPS (black) and HSE06 density of states (red) for (a) AgCuS and (b) Ag_3_CuS_2_. Valence band edge has been normalized to 0 eV.

Additionally, the XPS at the valence band edge was also recorded and compared to the calculated density of states diagrams, shown in Fig. 6. In both cases, the DoS is scaled using atomic orbital photoionisation cross-sections^71^ and the Gaussian smearing is adjusted to simulate experimental broadening. The major features of each XPS are matched well in the DoS, further indicating that the HSE06 functional predicts even the highly-correlated Cu and Ag states successfully.

### 3.4 Optical properties

**Fig. 7:**
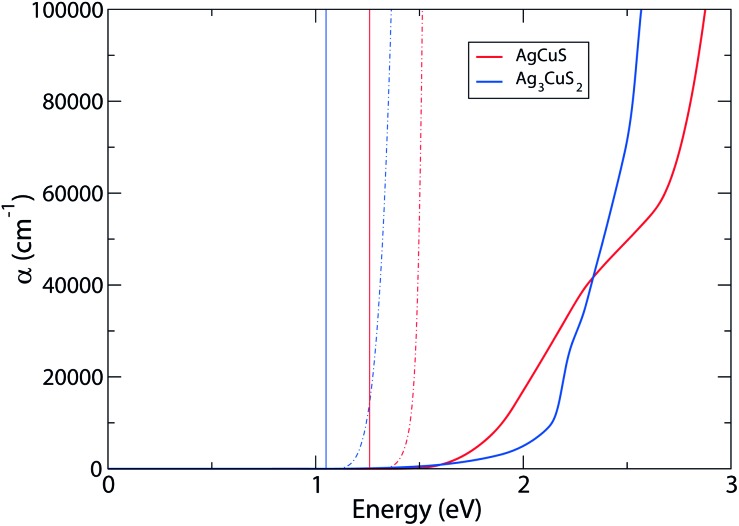
Calculated optical absorption of AgCuS and Ag_3_CuS_2_, with: absorption coefficients marked as bold lines; fundamental band gaps marked by vertical lines; (*αhν*)^2^, representative of the predicted optical band gap, is marked as alternating dot-and-dash lines.

As noted above, strong optical absorption is also necessary for good photovoltaic performance and so to further assess the suitability of these compounds, the attenuation coefficient, *α*, of both materials was calculated through the dielectric tensor.^56^ The resultant plot of *α* against energy is plotted in Fig. 7. These results show that the predicted optical gaps, given by (*αhν*)^2^, are ∼0.2 eV above the fundamental gaps for both materials. It should be remembered that our calculations are performed athermally, and we anticipate that the effects of temperature in the experimental measurements will lead to some lattice expansion and resultant shrinkage of the band gap, hence the fortuitous agreement between fundamental calculated and experimental optical band gaps in this report. Another source for this discrepancy may be the presence of defects, which were not accounted for in the optical calculations. Strong absorption, characterized by *α* > 10^4^, from both materials is also above that of the fundamental band gap – this is due to the VBM-CBM transition being symmetry forbidden in both cases. As a result, the lowest direct transition for AgCuS is increased to 1.46 eV, which is still within the suitable range for photovoltaic applications. This transition is marked in Fig. SF8 in the ESI,† originating from the band below the VBM, which is similarly dominated by Cu d and S p orbitals, with very little Ag contribution. On the other hand, Ag_3_CuS_2_ has multiple symmetry disallowed transitions, with the lowest direct allowed transition at 2.01 eV, indicating it could be a significantly poorer candidate for devices. This is reflected in the predicted absorption coefficient: for AgCuS, *α* increases relatively smoothly from ∼0.3 eV above the band gap, while the absorption for Ag_3_CuS_2_ remains low (<10^4^ cm^–1^) until above 2 eV. The target band for the transition in Ag_3_CuS_2_, as marked in Fig. SF8,† is also dominated by Cu s states, unlike the Cu d at the CBM.

To supplement this analysis, we have calculated the ‘spectroscopic limited maximum efficiency’ (SLME), a metric proposed by Yu and Zunger^72,73^ for assessing the theoretical maximum efficiency for both compounds, taking into account the nature of the band gap and the effect of the absorption, rather than the band gap alone. The SLME approach differs from the traditional SQ balance limit in two respects: first, it accounts for non-radiative recombination by treating the fraction of radiative electron–hole recombination current (*f*_r_) as *f*_r_ = e^–*Δ*/*k*_B_*T*^ where *k*_B_ is the Boltzmann constant, *T*, the temperature, and *Δ* is the difference between the lowest direct allowed transition and the fundamental band gap (*E*_g_); as such, an absorber with higher *Δ* is expected to perform worse due to the greater non-radiative loss. Second, rather than taking the absorptivity, *a*(*E*), as a step-wise function, with 0 below the band gap and 1 above, in SLME, it is a function of both absorption coefficient, *α*(*E*), and a thin film thickness, *L*: *a*(*E*) = 1 – e^–2*α*(*E*)*L*^. This additional assessment makes it particularly useful for screening potential photovoltaic materials, as it can identify materials that, while possessing an apparently suitable band gap according to the SQ limit, will be hampered by optical losses in real-world PV applications. Using a suitable film thickness of 2 μm, the SLME of AgCuS is 20.6%, above the threshold Yu and Zunger indicated as ‘high-SLME’ (extrapolating to infinite thickness gives an absolute maximum of 27%), while for Ag_3_CuS_2_, it is only 0.2%. The low SLME of Ag_3_CuS_2_ is likely to occur due to a combination of a much larger *Δ* and a low absorption coefficient, both of which reduce efficiency. It is possible that the symmetry-forbidden transitions are made more likely at room temperature due to thermal effects, such as lattice expansion or defects disrupting the crystal symmetry, however the large number of these in Ag_3_CuS_2_ may cause major problems for its future application in solar cells, as indicated by the vast difference in SLME. This difference, compared with the similar theoretical efficiencies predicted by the SQ limit, highlights the need to move beyond the use of band gaps as the primary metric for screening potential PV materials.

### 3.5 Electronic properties

The charge transport properties of these compounds can be just as crucial to the construction of an efficient solar cell. To this end, the resistivity of AgCuS was measured; Hall effect measurements were also possible on the AgCuS pellet, allowing for the measurement of its carrier mobility. A comparison of these measurements with previously recorded values for champion third-generation absorbers CZTS and methylammonium lead iodide (MAPI) is shown in Table 2. The positive Hall coefficient of AgCuS is indicative of p-type conductivity, which agrees with the conductivity measurements of the orthorhombic phase performed by Guin *et al.* ^39^

**Table 2:** Electronic behaviour of AgCuS, compared to other photovoltaic materials (stoichiometric, bar *, where S content ranges from 0–90%, and Cu, Zn and Sn ratios are variable)

Material	Hall coefficient (cm C^–1^)	Bulk concentration (cm^–3^)	Mobility (cm^2^ V^–1^ s^–1^)	Resistivity (Ω cm)
AgCuS	0.2304	1.7 × 10^18^	2.237	1.678
CZTS^74^	—	8 × 10^18^	6.0	0.13
CZTS^75^	160	3.9 × 10^16^	30	5.4
CZT(S,Se)*^76^	—	1.73 × 10^16^ to 1.4 × 10^15^	0.46–1.32	774–3300
MAPI^77^	—	∼10^9^	66	5.1 × 10^7^
MAPI^78^	—	2.8 × 10^17^	3.9	—

It was mentioned above that the effective masses of AgCuS are particularly low in some directions, close to those of CZTS; indeed, the mobility is the same magnitude as that of lower measurements of both CTZS and MAPI, which is as predicted, and very encouraging for its potential as an absorber layer in PV. In addition, as the pellet tested was pressed from a powder, and hence we might expect many grain boundaries and other defects, the measured mobility may be even higher for a stoichiometric thin film of AgCuS. Additional defects from our exploratory synthesis attempt may also be the cause of the relatively high carrier concentration in comparison to the device-quality values listed in Table 2; semiconductor-grade film growth may see this reduced as well. Previous work by Guin *et al.* calculated vacancy formation energies in AgCuS with PBE+U, finding *V*_Cu_ = +0.88 eV per formula unit.^39^ This is comparable to *V*_Cu_ = +0.77 eV found in CZTS^20^ and suggests that while such defects may be present at a reasonable concentration, their impact could be minimised with careful synthetic control. Antisite cation disorder of Ag and Cu in AgCuS is likely to be benign, however, as both cations are in the 1+ oxidation state. As such, a complete study of the defects in AgCuS at a high level of theory could be a worthwhile area for future study.

### 3.6 Band alignment

**Fig. 8:**
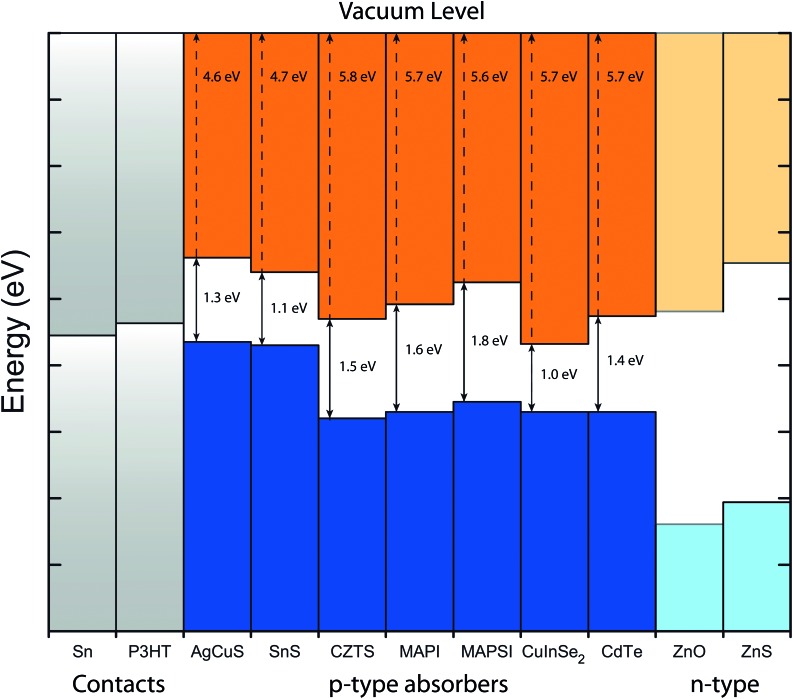
Valence band alignment of AgCuS with a number of other materials. Ionisation potentials and workfunctions of other materials have been taken from the literature.^28,79–84^

These results so far clearly indicate that AgCuS is a better candidate for photovoltaic applications than Ag_3_CuS_2_, with a more ideal band gap, stronger absorption onset and higher SLME. Thus, in order to aid in any further work towards AgCuS as a photoabsorber, its valence band alignment with vacuum (ionisation potential) was calculated using the core-level alignment approach.^57^ This method has been used with other chalcogenide absorbers^54^ and MAPI,^79^ with particular success at assessing suitable hole-transporting and buffer layers for photovoltaic absorber materials. To this end, the alignment of AgCuS, in comparison with a number of other p-type solar absorber materials, plus some n-type and contact layers, is shown in Fig. 8.

Like SnS, it is clear that AgCuS has a much lower (∼1 eV) predicted ionisation potential (IP) than other common photovoltaic absorber materials, including CZTS, which also has a valence band comprised primarily of Cu d and S p states. One possible reason for this may be the unusual trigonal planar coordination of the copper atoms in the AgCuS structure, compared to tetrahedral coordination in kesterite. Indeed, structural distortion has been proposed as the reason for the IP of SnS,^80^ and coordination environment is known to have an effect on the local Madelung potential of atoms in crystals,^85,86^ resulting in changes in the VB position.^11^ As a result of this difference in IP, the valence band level closely matches to the workfunctions of the organic conductor P3HT, and also Sn metal as buffer layers/contacts. On the other hand, ZnO, as used in Liu *et al.*'s Ag_3_CuS_2_ cell,^41^ has a large offset to the conduction band level of AgCuS, and we might expect would work poorly in a heterojunction cell with AgCuS. Instead, the related ZnS provides a much better match, with a conduction band level only ∼0.1 eV below that of AgCuS. From these results, it may be anticipated that, for example, Sn/AgCuS/ZnS/FTO may show particular promise as a potential cell architecture in future work.

## 4 Conclusion

In this study, we have examined two of the silver copper sulfides experimentally and theoretically, in the context of photovoltaics. While both compounds, AgCuS and Ag_3_CuS_2_, are expected to demonstrate very conducive electronic structures for PV applications, including low carrier effective masses and suitable, direct band gaps, the optical behaviour and SLME of Ag_3_CuS_2_ indicates that it will be severely limited by optical losses when used as an absorber. AgCuS possesses a more ideal band gap of 1.25 eV, observed theoretically and experimentally, good carrier mobilities and is predicted to exhibit a higher SLME and stronger optical absorption, and so it is anticipated to be a much more viable candidate for further study into the use of these materials in photovoltaics.
