# Supplementary material for: An assessment of silver copper sulfides for photovoltaic applications: theoretical and experimental insights
Source: J Mater Chem A Mater. 2016 Jul 23;4(32):12648–57. doi: 10.1039/c6ta03376h (PMC5059790; doi:10.1039/c6ta03376h)
Supplement: Supplementary file 1 [file TA-004-C6TA03376H-s001.pdf]

Electronic Supplementary Material (ESI) for Journal of Materials Chemistry A.
This journal is © The Royal Society of Chemistry 2016

Supplementary Information: An Assessment of Silver Copper Sulfides for Photovoltaic Applications: Theoretical and Experimental Insights

Christopher N. Savory,[†] Alex M. Ganose,[†,‡] Will Travis,[¶] Ria Atri,[¶] Robert G. Palgrave,[¶] and David O. Scanlon[*,†,‡]

†*University College London, Kathleen Lonsdale Materials Chemistry, Department of Chemistry, 20 Gordon Street, London WC1H 0AJ, UK*

‡*Diamond Light Source Ltd., Diamond House, Harwell Science and Innovation Campus, Didcot, Oxfordshire OX11 0DE, UK*

¶*University College London, Department of Chemistry, London WC1H 0AJ, UK*

E-mail: d.scanlon@ucl.ac.uk

Additional theoretical methods

Further functionals were used for comparison - these include PBEsol,[1] a functional specifically adapted for solids, and PBE0.[2] PBEsol, in particular, has been noted to provide very accurate lattice parameters for solid semiconductor systems in the authors' previous work.[3,4] In addition, a Hubbard U parameter of 5.17 eV was introduced in the 'PBEsol+U' calculations to partly counteract the incorrect delocalization of strongly correlated d electrons usually found in DFT calculations: due to the self interaction error inherent in DFT, there is larger than expected electron-electron repulsion, favouring the delocalization of electrons

to reduce this – this effect is particularly significant with the highly localized d orbitals in Ag and Cu. The addition of the U parameter through the rotationally invariant method by Dudarev *et al.* provides and electronic penalty against this, and leads to a more accurate assessment of the electronic structure.[5]

Geometrical Optimization

Supplementary Table ST 1: Calculated lattice parameters of AgCuS, percentage difference from experiment or experimental error in brackets. All cell angles were found to be 90°

	a (Å)	b (Å)	c (Å)
PBEsol	3.9262 (-3.37%)	6.6847 (+0.85%)	8.0361 (+0.81%)
PBEsol+U	3.9489 (-2.81%)	6.5864 (-0.63%)	7.9567 (-0.18%)
HSE06	4.0422 (-0.52%)	6.7522 (+1.87%)	8.4311 (+5.77%)
PBE0	4.0399 (-0.58%)	6.7404 (+1.69%)	8.4181 (+5.61%)
Experiment[6]	4.0633(2)	6.6281(4)	7.9713(4)

Firstly, AgCuS was considered, and the results of the structure optimization calculations are displayed in Supplementary Table 1. From these results, it is apparent that both PBEsol and PBEsol+U underestimate the a lattice parameter compared to the experimental value, but the values for b and c are overestimated by PBEsol to a lesser extent than with a, while intoducing a Hubbard U parameter in PBEsol+U, causes b and c to be underestimated, with all parameters closer to experiment than PBEsol. HSE06 and PBE0 however give values for a that are much closer to experiment than the GGA functionals, but are further away in the others, especially c, where both overestimate the experimental value by greater than 5%. As a result, it is difficult to conclude which method describes the structure more accurately and so the electronic calculations for each functional were performed from the corresponding optimized structure. The authors note that these differences appear large, indeed they are noticeably larger than usually found with benchmark functionals such as PBEsol. With AgCuS in particular having a low temperature phase transition,[7] and predicted to be soft, with many low energy phonon modes,[8] it is possible that the effect of temperature on the lattice

parameters is significant, leading to these differences between DFT-relaxed and experimental structures.

Supplementary Table ST 2: Calculated lattice parameters of Ag_3CuS_2, percentage difference from experiment or experimental error in brackets. All cell angles were found to be 90°

$I4_1/amd$	a (Å)	c (Å)
PBEsol	8.2279 (-4.85%)	12.2428 (+3.86%)
PBEsol+U	8.2578 (-4.51%)	12.1745 (+3.28%)
HSE06	8.8350 (+2.17%)	11.8013 (+0.11%)
PBE0	8.7969 (+1.73%)	11.8239 (+0.30%)
Experiment[9]	8.6476(5)	11.7883(8)

Unlike with AgCuS, here we see a distinct difference between the accuracy of the GGA and hybrid functionals at describing the crystal structure. With the $I4_1/amd$ phase of Ag_3CuS_2, the GGA functionals underestimate the a lattice parameter and overestimate c, both of which are by a significant amount (greater than ±3%) when compared to experiment. The PBEsol+U method shows an improvement on PBEsol, but that improvement is small – around 0.4% closer to the experimental values, and similar to the difference seen with AgCuS. A much larger improvement comes with moving to HSE06 and PBE0 – both do overestimate the value of a, but by less than 2.5%, and their values of c show very good agreement with experiment.

Electronic Figures

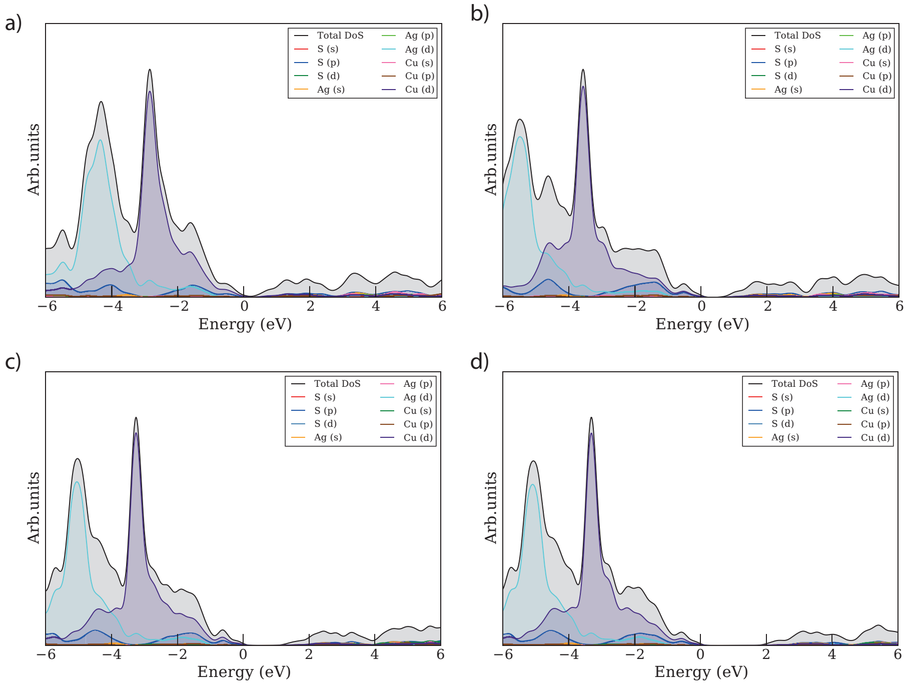

Supplementary Figure SF 1: Total and Partial Density of States diagrams of AgCuS, using a) PBEsol, b) PBEsol+U, c) HSE06 and d) PBE0; individual partial DoS are labelled in legends, Energy = 0 eV is set to valence band maximum.

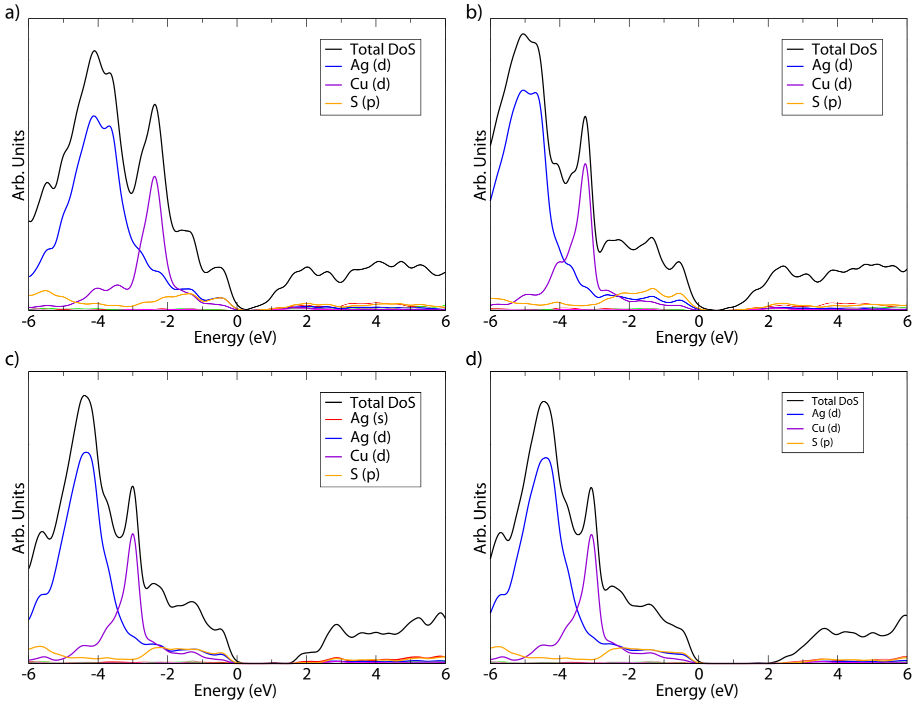

Supplementary Figure SF 2: Total and Partial Density of States diagrams of RT-Ag_3CuS_2, using a) PBEsol, b) PBEsol+U, c) HSE06 and d) PBE0; individual partial DoS are labelled in legends, Energy = 0 eV is set to valence band maximum.

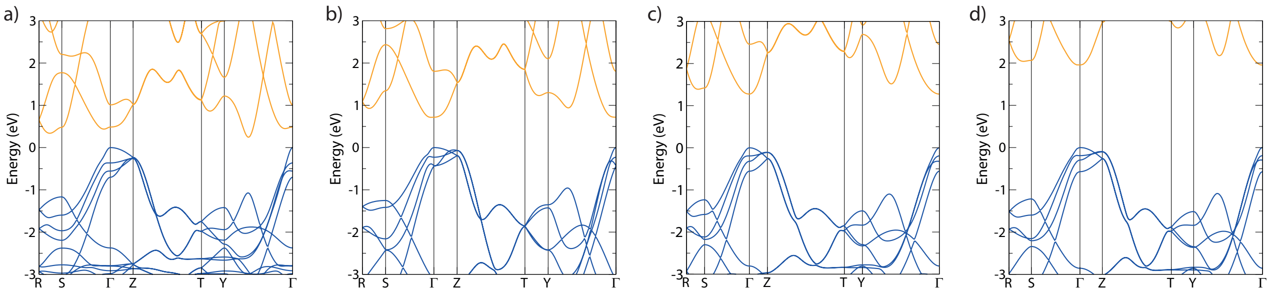

Supplementary Figure SF 3: Band structure diagrams of AgCuS, using a) PBEsol, b) PBEsol+U, c) HSE06 and d) PBE0; valence band marked in blue, conduction band marked in orange, Energy = 0 eV is set to valence band maximum.

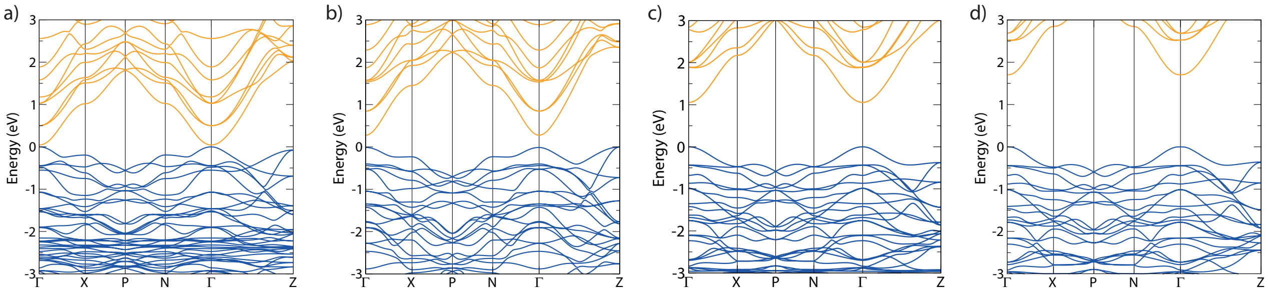

Supplementary Figure SF 4: Band structure diagrams of RT-Ag_3CuS_2, using a) PBEsol, b) PBEsol+U, c) HSE06 and d) PBE0; valence band marked in blue, conduction band marked in orange, Energy = 0 eV is set to valence band maximum

XRD and XPS

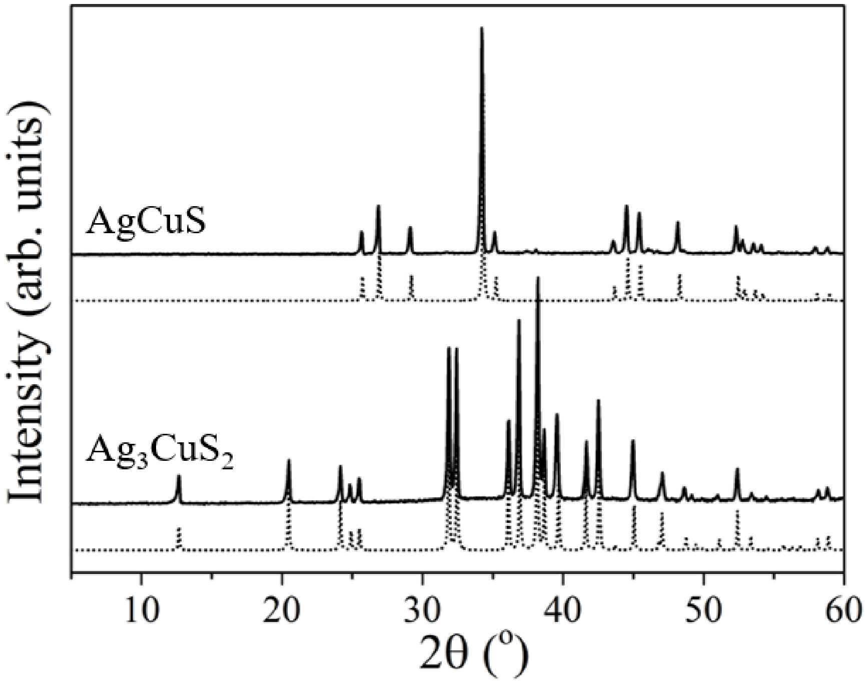

Supplementary Figure SF 5: X-ray diffraction patterns of AgCuS and Ag3CuS2: solid lines are recorded patterns, dotted lines are patterns simulated from previous crystal structures.[6,9]

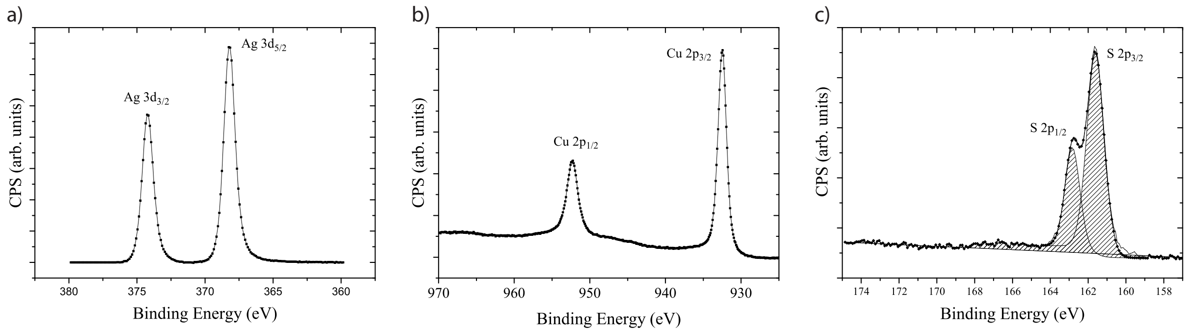

Supplementary Figure SF 6: XPS core levels of Ag 3d, Cu 2p and S 2p in as-synthesised powder of AgCuS.

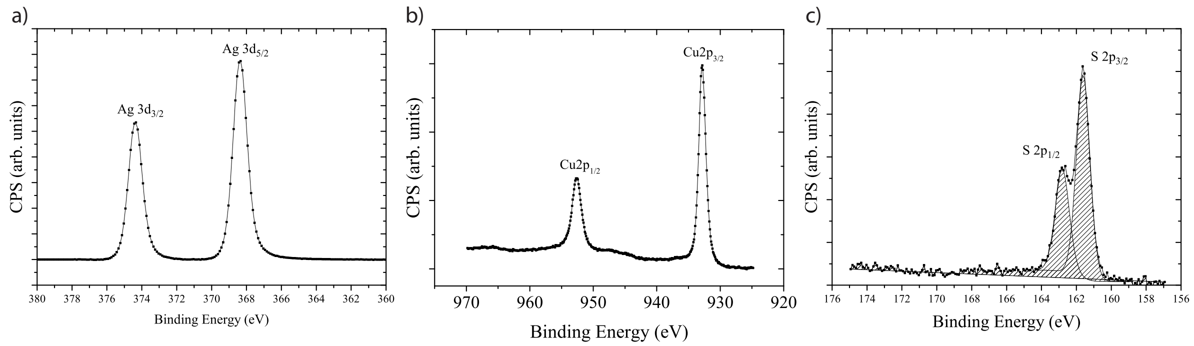

Supplementary Figure SF 7: XPS core levels of Ag 3d, Cu 2p and S 2p in as-synthesised powder of Ag_3CuS_2.

Optical Behaviour

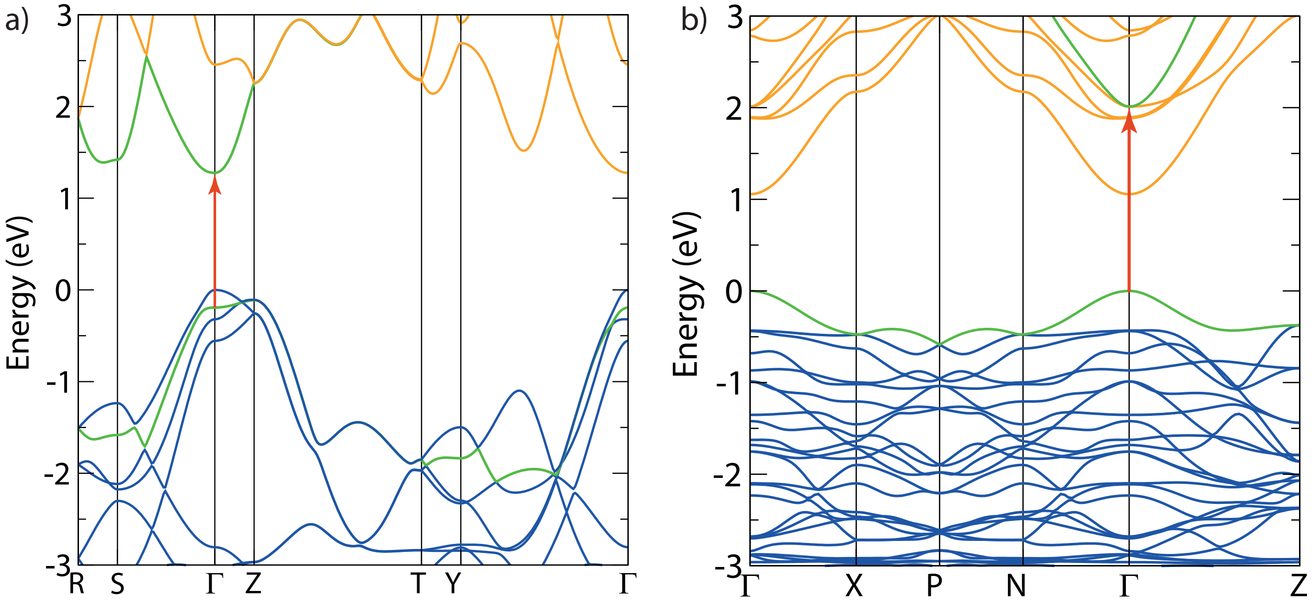

Supplementary Figure SF 8: Band structures of a) AgCuS and b) Ag_3CuS_2, with the lowest direct allowed transition marked and the bands involved coloured green.

References

(1) Perdew, J. P.; Ruzsinszky, A.; Csonka, G. I.; Vydrov, O. a.; Scuseria, G. E.; Constantin, L. A.; Zhou, X.; Burke, K. *Physical Review Letters* **2008**, *100*, 136406.

(2) Adamo, C.; Barone, V. *The Journal of Chemical Physics* **1999**, *110*, 6158.

(3) Savory, C. N.; Palgrave, R. G.; Bronstein, H.; Scanlon, D. O. *Scientific Reports* **2016**, *6*, 20626.

(4) Ganose, A. M.; Butler, K. T.; Walsh, A.; Scanlon, D. O. *Journal of Materials Chemistry A* **2016**, *4*, 2060–2068.

(5) Dudarev, S. L.; Botton, G. A.; Savrasov, S. Y.; Humphreys, C. J.; Sutton, A. P. *Physical Review B* **1998**, *57*, 1505–1509.

(6) Trots, D. M.; Senyshyn, A.; Mikhailova, D. A.; Knapp, M.; Baehtz, C.; Hoelzel, M.; Fuess, H. *Journal of Physics: Condensed Matter* **2007**, *19*, 136204.

(7) Baker, C. L.; Lincoln, F. J.; Johnson, A. W. S. *Acta Crystallographica Section B Structural Science* **1991**, *47*, 891–899.

(8) Guin, S. N.; Pan, J.; Bhowmik, A.; Sanyal, D.; Waghmare, U. V.; Biswas, K. *Journal of the American Chemical Society* **2014**, *136*, 12712–12720.

(9) Trots, D. M.; Senyshyn, A.; Mikhailova, D. A.; Vad, T.; Fuess, H. *Journal of Physics: Condensed Matter* **2008**, *20*, 455204.
